# New-Generation Washing Agents in Remediation of Metal-Polluted Soils and Methods for Washing Effluent Treatment: A Review

**DOI:** 10.3390/ijerph17176220

**Published:** 2020-08-27

**Authors:** Zygmunt M. Gusiatin, Dorota Kulikowska, Barbara Klik

**Affiliations:** Department of Environmental Biotechnology, Faculty of Geoengineering, University of Warmia and Mazury in Olsztyn, 10-719 Olsztyn, Poland; dorotak@uwm.edu.pl (D.K.); barbara.klik@uwm.edu.pl (B.K.)

**Keywords:** soil washing, soil flushing, heavy metals, soil remediation, treatment of spent washing solution

## Abstract

Soil quality is seriously reduced due to chemical pollution, including heavy metal (HM) pollution. To meet quality standards, polluted soils must be remediated. Soil washing/soil flushing offers efficient removal of heavy metals and decreases environmental risk in polluted areas. These goals can be obtained by using proper washing agents to remove HMs from soil. These washing agents should not pose unacceptable threats to humans and ecosystems, including soil composition. Currently, it is desirable to use more environmentally and economically attractive washing agents instead of synthetic, environmentally problematic chemicals (e.g., ethylenediaminetetraacetic acid (EDTA)). The usefulness of novel washing agents for treatment of heavy metal-contaminated soils is being intensively developed, in terms of the efficiency of HM removal and properties of washed soils. Despite the unquestionable effectiveness of soil washing/flushing, it should be remembered that both methods generate secondary fluid waste (spent washing solution), and the final stage of the process should be treatment of the contaminated spent washing solution. This paper reviews information on soil contamination with heavy metals. This review examines the principles and status of soil washing and soil flushing. The novel contribution of this review is a presentation of the sources and characteristics of novel washing agents and chemical substitutes for EDTA, with their potential for heavy metal removal. Methods for treating spent washing solution are discussed separately.

## 1. Introduction

Soils polluted with heavy metals (HMs) occur at numerous sites throughout the world. This is a result of different anthropogenic activities such as mining, smelting, warfare and military training, electronic industries, fossil fuel consumption, waste disposal, agrochemical use, and irrigation [1]. Kabir et al. [2] listed the main types of industries that affect HM abundance in soils: (i) mining, smelting, and metallurgical, (ii) chemical and petrochemical, (iii) textile, (iv) leather, and (v) non-metallic mineral, especially cement. In mining and smelting sites, mainly Cu, Cd, Pb, and Zn are detected, whereas, around chemical and petrochemical plants, Cd, Pb, Cr, and As are the most abundant. Due to their non-biodegradability, toxicity, persistence, and bioaccumulation in the food chain, HMs are considered priority pollutants in the environment [3]. Reduced crop productivity is also a consequence of soil HM pollution [4]. 

HMs commonly present in soils include Al, Cd, Cu, Co, Cr, Hg, Ni, Mn, Pb, and Zn [5]. Of these HMs, As, Pb, Hg, Cd, and Cr (VI) are ranked first, second, third, seventh, and 17th, respectively, of the 275 substances on the Substance Priority List of the Agency for Toxic Substances and Disease Registry [6]. Soil pollution with HMs is a global problem. Over 10 million contaminated sites were identified in the world, of which more than 50% are contaminated with HMs and/or metalloids [5]. The highest soil contamination levels occur in developed countries, i.e., the United States of America (USA), Australia, Germany, Sweden, and China. For example, around 600,000 ha of soil is contaminated with HMs in the USA, with Cd, As, and Pb as the most prevalent [7]. The main source of soil pollution in the USA is the mining industry. There are about 14,500 active coal, metal, and nonmetal mineral mines and approximately 13,000 abandoned coal mines [8]. In Europe, industrial production, municipal waste treatment/disposal, and the oil industry account for 41.4%, 15.2%, and 14.1% of soil pollution, respectively. There are potentially over three million polluted sites, of which about 8% are sites polluted with HMs. HMs constitute about 38% of all identified pollutants in European soils [9]. A high soil contamination with HMs was also reported for China. About 25% of total arable farmland area in China (>80 million ha) is contaminated by Pb, Cd, Cr, Sn, and Zn [5]. Example concentrations of HMs in soils in the vicinity of industrial plants are given in Table 1.

Excessive HM concentrations in soil, especially in the case of multiple HMs, lead to unfavorable changes in soil quality. Contaminated soils must be excluded from agricultural use and they pose a risk of groundwater contamination. Thus, it is highly desirable to apply suitable remedial approaches to polluted soil, which can reduce the risk of HM pollution. Two suitable remediation technologies are soil washing and soil flushing. 

The main purpose of this review is to provide a comprehensive overview of soil washing and soil flushing methods, as well as novel washing agents (WAs) as substitutes of conventional WAs, and their applicability for removing HMs from soil. In addition, the methods of treatment of spent washing solutions (SWS) are presented. 

## 2. Principles of Soil Washing and Soil Flushing

Soil washing is a physical and/or chemical process that aims to effectively remove pollutants from soil. Soil washing can be conducted in one of three variants: physical separation, chemical extraction, or physical separation followed by chemical extraction [22]. Physical separation aims to separate the most contaminated particles from bulk soil and reduce the volume of polluted soil to be treated. At this stage, soil washing with water allows the coarse-grained particles to separate from the polluted fine particles. Chemical extraction is based on pollutant solubilization with a washing solution (water with chemical additives) and their transfer from soil particles into the washing solution [23]. 

Generally, soil washing based on physical separation and chemical extraction includes six steps [24,25,26]:

- Pretreatment (removal of oversized materials from soil),
- Separation between coarse- and fine-grained soil particles in a scrubbing unit (separation of the coarse-grained soils is commonly done by using mechanical screening such as trommels, while the fine-grained soils are sorted using hydrocyclones or other methods),
- Coarse-grained treatment (usually coarse-grained soil particles >0.05 mm are likely unpolluted or minimally polluted; thus, they can be treated using surface attrition or washing with water),
- Fine-grained treatment (because fine soil particles <0.05 mm are highly contaminated, they should be treated with a suitable washing solution containing water and chemicals using sonication or mechanical agitation),
- SWS treatment (treatment of SWS from washing coarse and fine soil particles; necessary to reuse washing solutions or to dispose of them in sewers),
- Residual management (residual materials, i.e., treated soil, and the sludge of dispersed fine particles output during soil washing; if residuals are still considered polluted, they may require further treatment before disposal).

**Figure 1:**
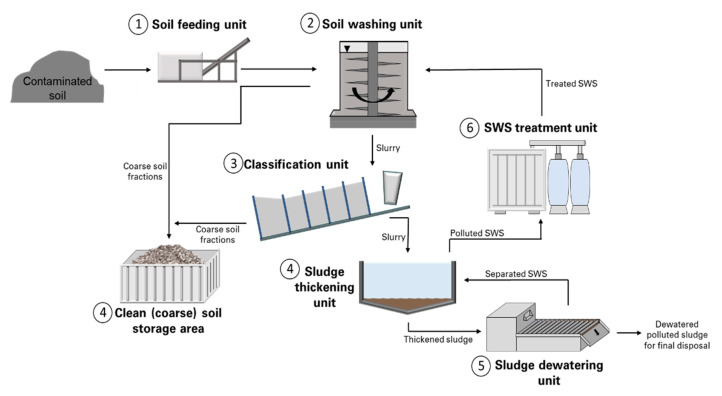
Soil washing system (adapted from www.dekonta.cz [27]). The numbers (1–7) indicate the steps of soil washing.

Overall, there are many different soil washing systems that were developed, and the systems can vary from site to site due to location-specific constraints for soils or pollutants. In Figure 1, an example of DEKONTA’s soil washing system is presented [27].

**Figure 2:**
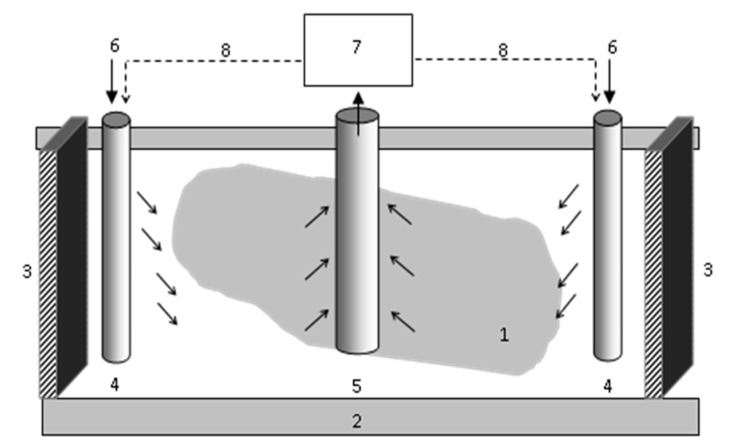
In situ soil flushing: polluted ground (1), low permeable zone (2), physical barrier (3), injection well (4), extraction well (5), washing solution (6), spent washing solution (SWS) treatment module (7), reinjection of treated SWS (8).

In contrast to soil washing, soil flushing treats the soil in situ using an injection/recirculation system (Figure 2). The process begins with the drilling of injection wells and extraction wells into the ground, where the pollutants were found, and the SWS treatment system must be mobile or built on site [28]. The washing solution is introduced to the soil with pumps through injection wells or through an infiltration process when pollutants are present in shallow layers of the ground. As the solution passes through the polluted area, the soil pollutants are mobilized by solubilization or chemical interactions. Next, the pollutant-bearing solution and groundwater are pumped out via extraction wells, and then brought to the surface for disposal, recirculation, or on-site treatment and reinjection [29,30,31]. Soil flushing is most appropriate for soils with high hydraulic conductivity [22].

Both soil washing and soil flushing work with water or washing solutions (water with chemicals). Depending on the type of technology, water or washing solutions can be used (i) to disaggregate the treated soil, (ii) to suspend the soil particles in separation equipment, or (iii) to dissolve or to solubilize pollutants in soil [23]. Soil washing can consume more water than soil flushing, and its operational costs are higher. Thus, the use of water or water with additives should be effectively managed, not only to reduce the overall amount of water used during processing, but also to ensure that any pollutants transferred into the water during the process do not re-contaminate clean products [23]. 

Soil washing is a well-established technology of soil remediation in many European countries (the Netherlands, Germany, Belgium, Switzerland), as well as in North America and Japan [23]. According to Dermont et al. [22], among 37 field soil washings performed in 1989–2007, 43% were based on physical separation, 8% were based on chemical extraction, and 47% were based on physical separation and chemical extraction. Full-scale soil washing plants exist as centralized permanent treatment sites, where polluted soil is brought to the plant, or as mobile/transportable units, where polluted soil is treated on the site. 

In the Netherlands, there are five permanent soil washing sites (BMR Schiedam, SBD Amersfoort, BVNN BKD Groningen, GBD Alkmaar, and BVNN BKD Heerenveen) where asbestos-contaminated soil, dredged sediment, sand from sewers, contaminated roof gravel, contaminated granulate, and stony materials are processed [32]. Soil washing is used at many large-scale cleanup sites in the USA, including Miami Harbor, Fox River (Wisconsin), and the King of Prussia Superfund Site [33]. Mobile soil washing systems are modular construction units that can be deployed at different sites. The USA and some European countries (e.g., Sweden) developed mobile soil washing systems in the 1980 s and employed the devices in field sites to clean up contaminated soils [1,34]. A pilot-scale mobile soil washing system was proposed by Ko et al. [35] to treat soil polluted with As, Zn, and Ni coming from an iron mining area in Korea. The system consisted of a soil washing scrubber (drum type equipped with an inner screw blade to mix soil with washing solution), a vibrating screen, a screw feeder, a high-pressure air supply (to provide mechanical turbulence between soil and washing solution), and a ceramic filter. The mass of soil treated with individual washing solutions (HCl, H_2_SO_4_, or H_3_PO_4_) in this mobile system was 40 kg [35]. Many environmental companies offer soil washing systems. Global leaders in the field of large-scale washing of soils are Boskalis Environmental [36] and ART Engineering LLC [37]. 

Soil flushing is less applicable than soil washing. So far, it was only selected for soil flushing in Superfund sites in the USA to remove volatile and semi-volatile organic compounds and HMs from Lipari Landfill in New Jersey, HMs from a chrome plating area in Oregon, and HMs from a pesticide manufacturing area at the Vineland Chemical Company in New Jersey [28]. 

Soil washing and soil flushing differ in applicability, soil treatment, limitations, and costs. Both technologies are compared in Table 2.

## 3. Novel WAs and Their Applicability for HMs Removal from Soil

Both soil washing and soil flushing rely on solubilization and mobilization of HMs by altering soil acidity, solution ionic strength, redox potential, or complexation [1]. The type of WA has the largest effect on the efficiency of soil treatment. An ideal washing solution should considerably improve the solubility and mobility of HMs and simultaneously interact weakly with soil constituents. Among commonly used WAs, there are mineral acids (e.g., HCl, H_2_SO_4_, HNO_3_), low-molecular-weight organic acids (e.g., oxalic and citric acids), synthetic organic chelating agents (e.g., ethylenediaminetetraacetic acid (EDTA), diethylenetriaminepentaacetic acid (DTPA), and ethylenediaminedisuccinic acid (EDDS)), and biosurfactants (e.g., saponin, rhamnolipids) [39]. Although these agents have a high extraction efficiency, some of them can cause ecological problems in the soil environment. Acids can change the soil structure, decrease soil productivity via massive loss of nutrients and organic matter, and can be toxic to soil microorganisms. EDTA can be toxic, persistent, or slowly transformed in the environment. Biosurfactants, similarly to synthetic organic chelating agents, are effective in HM removal from soil, but their price is still high. Soil washing/flushing can be a more attractive option if the WAs are nontoxic, biodegradable, and easily available. Therefore, efforts should be directed toward the development of low-cost and environmentally friendly WAs, which can be used as substitutes for conventional WAs. 

### 3.1. Sources of New-Generation WAs

Novel WAs proposed for HM removal from soils are green in nature. Most of them are isolated or extracted from geochemical resources, organic waste materials, composts, and others. These WAs include dissolved organic matter (DOM), sometimes called natural organic matter (NOM), and soluble humic substances (SHS). The utilization of these WAs is a new form of biodegradable waste management, compatible with the principle of sustainable development. It is worth mentioning that WAs intended to be used at the technical scale of soil washing/soil flushing should be effective, safe, relatively cheap, and available. The main sources of next-generation WAs are briefly described below. 

#### 3.1.1. Geochemical Resources

*Leonardite* is highly oxidized lignite, and it contains much higher contents of humic acids (HAs) (about 15%) than compost, manure, straw, etc. (below 1%) [40,41]. Leonardite is a source of soluble HAs [42] and commercially available humic substances (HS) (e.g., Powhumus WSG-85) [41] and non-commercially available HS, which are directly extracted from lignite [43]. HS extracted using leonardite was shown to be a low-cost and highly effective natural surfactant for treatment of industrial soils [44]. 

#### 3.1.2. Unprocessed Wastes

Wastes are an important source of naturally occurring dissolved organics that can be used to remove HMs from soil. The use of wastes as a source of WAs is a part of the circular economy, in which wastes serve as a resource for producing new products [45]. The main waste materials that serve as sources of novel WAs are municipal sewage sludge and agro-industrial wastes.

*Municipal sewage sludge* is an organic by-product from wastewater treatment that can be used as fertilizer and as feedstock for composting and biogas production. The amount of sewage sludge is gradually increasing around the world, and sludge landfilling is highly limited or forbidden in many countries. Thus, there is a need to employ new methods for its disposal and utilization.

Sewage sludge contains high levels of organic matter, nitrogen, phosphorus, and micronutrients. In addition, it may contain humus-like materials, because, during municipal wastewater treatment, mineralization of organic matter is accompanied or closely followed by humification [46]. According to Li et al. [47], raw sewage sludge from a full-scale municipal wastewater treatment plant (WWTP) contained 112.5 mg/g in total of HAs and fulvic acids (FAs). The HS extracted from sewage sludge accounted for ca. 15% of total solids and 27% of organic matter, and HAs were the main fraction of HS (91%). For comparison, sewage sludge from some wastewater treatment plants in Poland, operated as mechanical–biological systems, was characterized with a content of HS from 94 to 162 g/kg [46].

Sewage sludge can be a source of dissolved organic matter (DOM), soluble humic-like substances (HLS), and soluble humic substances (SHS) [46,48]. These WAs might simultaneously remove HMs and improve soil fertility after remediation [48].

*Agricultural wastes* from agro-industrial production can be used as a source of DOM to remove HMs from contaminated soils. Feng et al. [49] tested DOM extracted from pineapple peel, soybean straw, broad bean straw, and tea residue. Borggaard et al. [50] proposed that soluble natural HS prepared from (i) forest litter by centrifugation, filtration, and treatment with an H^+^-saturated cation-exchange resin or from (ii) cow slurry (aqueous suspension of feces, urine, and bedding), treated by oxidizing hydrolytic degradation at a specific temperature and pressure, can be attractive and promising alternatives to EDTA for remediation of soils contaminated with HMs.

#### 3.1.3. Processed Waste

*Wine-processing waste sludge* (WPWS), also known as distillery sludge, is a mixture of anaerobically digested sludge, activated sludge, and coagulated sludge. Sewage in the distillery is derived from the domestic wastewater of workers, rainwater, and washings of wine-making ingredients and wine-processing machines. As a result, the WPWS is devoid of any toxic HMs and can be used for soil remediation [51]. The WPWS contains organic matter (40–50%), nitrogen (1.6%), phosphorus (0.06%), calcium (5.9%), iron (17%), aluminum (1.7%), manganese (0.06%), sulfur (3.0%), and silicon (5.2%) [51,52]. WPWS can used as a source of DOM [53,54,55].

*Composted wastes* are an important source of HS. During composting, HS precursors are mainly formed during the heating and thermophilic phases, whereas HS are mainly polymerized during the cooling and maturing phases [56]. In addition, the content of FAs in HS decreases, whereas that of HAs increases. A high content of FAs can reflect a low degree of compost maturity and humification. There are many factors affecting the formation of HS during composting, such as the type of raw materials, their particle size, the presence of compost additives, microbial and enzymatic activity, and environmental conditions (temperature, pH, moisture, C/N ratio, oxygen content) [57]. For example, Gusiatin and Kulikowska [58] demonstrated that the content of HS in compost made from municipal sewage sludge and lignocellulosic materials increased from 98.5 g/kg to 114.4 g/kg as compost maturation time was extended from 3–12 months. Pérez-Esteban et al. [59] found that sheep and horse manure compost and pine bark compost were both rich in HS (5.4% and 6.2%, respectively), but the type of composted wastes affected the content and the composition of HS. The manure compost had a higher degree of humification than the pine bark compost due to a higher content of HAs in HS [59]. Similarly, Piccolo et al. [60] found that HS from compost had a greater degree of aromaticity, and more carboxyl and phenolic groups when the content of cow manure and maize straw in the feedstock was increased. Those authors concluded that the properties of compost may differ markedly depending on compost maturation and the type of biomass used in the composting process, while the molecular composition of the HS extracted from compost may vary as well. The HS extracted from different composts can serve as WAs in soil remediation projects. 

In addition to compost, the leachate produced during composting can also be a source of WAs. Chiang et al. [61] reported that a liquid fertilizer obtained via food-waste (chicken waste and vegetable debris) composting could be used to prepare a dissolved organic carbon (DOC) solution. The obtained liquid fertilizer was rich in organics and nutrients for plant growth.

*Digestate* is another option. Li et al. [47] found that, in digested sludge, the content of HS was 105.4 mg/g, and HAs were the major constituent of HS (84%). In addition, during anaerobic digestion of sludge, 16.3% of HAs and 27.0% of FAs were degraded, while a limited humification was observed [62].

*Humified straw* can be decomposed and release organics into the environment after being applied to soil, and the components released during the humification process will greatly affect the behavior of some soil pollutants [63]. On this basis, Fan and Zhang [64] proposed a simple method for producing an environmentally friendly solution of DOC derived from humified corn straw for soil remediation. The process of producing this solution included straw cleaning with purified water, its drying at 60 °C, and grinding to 1 mm. Then, the straw was humified with reclaimed water at 25 °C and 20 rpm without artificial lighting for 60 days. The humification process increased the concentration of total organic carbon (TOC), total nitrogen, and total phosphorus in the solution, while the pH remained neutral. 

WAs isolated from wastes possess multiple binding sites in the form of carboxylate and phenolate groups, and they have the ability to form highly stable and soluble organo-metallic complexes with HMs such as As, Cd, Cu, Ni, Pb, and Zn [65]. Organic wastes are rich not only in organic matter, but also in macroelements. Thus, the use of WAs recovered from these wastes might simultaneously remove HMs and improve the fertility of remediated soil. Wastes can be an attractive source of WAs as they are easily obtainable due to their large production all over the world. DOM for soil washing can also be obtained from soils [65]. Soil DOM consists mostly of FAs, with up to 10% low-molecular-weight organic acids such as acetic acid, benzoic acid, citric acid, lactic acid, malonic acid, oxalic acid, phthalic acid, salicylic acid, succinic acid, and tartaric acid [65,66,67].

**Figure 3:**
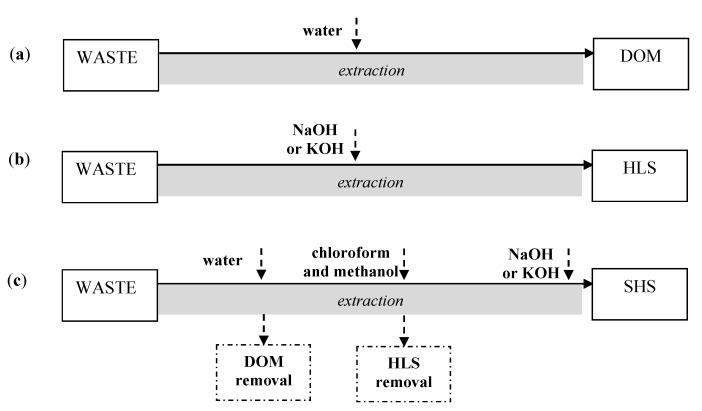
Isolation of washing agents (WAs) from wastes: (**a**) dissolved organic matter (DOM); (**b**) humic-like substances (HLS); (**c**) soluble humic substances (SHS).

The composition of DOM can vary, depending on the source and the type of extractant used for DOM recovery. In general, for isolation of specific soluble organics from wastes, water, or alkaline chemicals (KOH or NaOH) are used (Figure 3). With these extractants, three types of washing solutions can be obtained, i.e., DOM, HLS, and SHS. DOM isolated with water contains mainly low-molecular-weight organics and FAs. HLS contains both low-molecular-weight organics and macromolecular compounds (FAs and HAs), whereas, in SHS, FAs and HAs predominate.

In Table 3, WAs from different organic sources are presented. In batch soil washing experiments conducted with DOM, HLS, and SHS from waste sources, these WAs were highly effective for removing HMs and As from contaminated soils. The type of wastes and the conditions of extraction affect the concentration of the obtained WAs, which vary from below 0.5 to nearly 10 g TOC/L. In addition, the WAs isolated from wastes exhibit surface-active properties, as confirmed by their ability to reduce the surface tension of water, comparable with the ability exhibited by plant biosurfactants [68]. Reducing the surface and interfacial tension promotes the transfer of HMs from the soil to the solution, which affects the effectiveness of soil washing [69]. 

### 3.2. Examples of Chemical Substitutes for EDTA

*Synthetic humic-like acids* (SHLA). HAs, apart from natural sources, can also be synthetized through abiotic humification. This process is based on transformations of humic precursors (e.g., amino acids, sugars, and quinones) to HS in the presence of a metallic oxide (e.g., MnO_2_, Fe_2_O_3_, Al_2_O_3_), which catalyzes and enhances these transformations [70]. Yang and Hodson [71] found that SHLA prepared via abiotic humification, using catechol and glycine as humic precursors and an MnO_2_ catalyst, was characterized with higher Cu complexation ability than commercial HA (CHA). This is because SHLA is characterized with a higher number of acidic functional groups and *O*-alkyl functional groups than CHA.

*Potassium lignosulfonate* (KLS). During the production of wood pulp using sulfite pulping, KLS is generated as a by-product. KLS can be applied to agricultural fields as a soil conditioner and chelate fertilizer [39]. KLS is a water-soluble anionic polyelectrolyte polymer [72] with complexing ability and surface-active properties; thus, it has potential to be used in remediation of soils polluted with HMs. The KLS showed high efficiency of Cu (55–73%) and Pb (53–67%) removal in batch soil washing. In column leaching (soil flushing), the process removal efficiency decreased to 23–40% for Cu and 20–36% for Pb. KLS improved soil fertility by increasing the content of organic matter and available nitrogen/phosphorus/potassium (NPK) in soil.

**Table 3:** Selected properties of WAs isolated from wastes and their efficiency in HM removal from soils.

Type of WA	Source of WA	Selected Properties of Original WA			Soil Washing Efficiency (%) under Optimum Conditions						Ref.
		Concentration (g TOC/L)	pH	Surface Tension (mN/m)	As	Cd	Cu	Ni	Pb	Zn	
DOM	Municipal sewage sludge	6.8	6.9	45.0	–	–	57	–	5	39	[46]
	Corn straw	0.24	6.8	–	–	76	–	–	58	–	[64]
	Liquid fertilizer from food-waste composting	7.91	7.4	–	–	–	–	–	–	43	[61]
	Municipal sewage sludge	6.8	6.9	45.0	–	82–87	–	–	–	–	[48]
HLS	Wine-processing waste sludge	2.0	Alkaline	–	–	80	–	–	–	–	[54]
	Wine-processing waste sludge	2.5	12.0	–	–	–	–	–	86.5–93.0	–	[55]
	Municipal sewage sludge	9.7	11.7	40.4	–	79–81	–	–	–	–	[48]
SHS	Composted sewage sludge	4.1	11.9	42.5	18–27	–	–	–	–	–	[73]
	Leonardite	4.7	Alkaline	54.6	–	35.3–75.0	–	–	–	–	[43]
	Composted sewage sludge	2.2	12.7	51.7	–	79.1–82.6	51.5–71.8	35.4–46.1	44.8–47.6	27.9–35.8	[74]
	Composted sewage sludge	4.0	13.0	48.0	–	36.5–69.1	53.2–80.7	–	–	–	[75]
	Compost park and garden waste (as percolate)	100 *	6.0	–	16.0–61.0	–	61.0–95.0	–	–	–	[76]
	Municipal sewage sludge	5.0	12.3	42.4	–	75–80	–	–	–	–	[48]

* Expressed in mM, TOC is total organic carbon.

**Figure 4:**
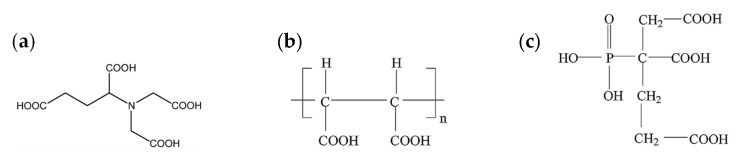
Structures of chemicals used for soil washing: (**a**) *N,N*-bis(carboxymethyl)-l-glutamic acid (GLDA); (**b**) hydrolytic polymaleic anhydride (HPMA); (**c**) 2-phosphonobutane-1,2,4-tricarboxylic acid (PBTCA).

*N,N-Bis(carboxymethyl)-l-glutamic acid* (GLDA) is a new kind of chelating agent, which can serve as a substitute for EDTA or EDDS (Figure 4a). GLDA is based on the food-approved natural amino acid salt, monosodium l-glutamate, which is produced via biochemical conversion of vegetable material (such as sugar beet waste) [77]. GLDA is highly soluble over a wide pH range and is non-toxic to ecosystems and more biodegradable (>80%) than EDTA and EDDS [78]. GLDA was employed for treatment of soils from mining and former non-ferrous HM refinery areas in China [78]. It showed the same efficiency of Cd and Zn removal from soils as EDTA, as well as high efficiency of Pb removal. In addition, GLDA solubilized less Al, Ca, Fe, Mn, and Mg from soils than EDTA, which positively affected revitalization of washed soils. GLDA can be easily regenerated from SWS and can be reused for soil washing. Interestingly, regenerated GLDA showed a similar efficiency of HM removal to fresh GLDA [78]. Thus, GLDA can serve as an alternative to EDTA for treatment of soils heavily polluted with HMs.

*Hydrolytic polymaleic anhydride (HPMA) and 2-phosphonobutane-1,2,4-tricarboxylic acid (PBTCA)*. HPMA is a non-toxic and water-soluble polymeride with a low molecular weight (Figure 4b). It is widely used in desalination plants, flash vaporization equipment, low-pressure boilers, steam locomotives, crude oil evaporation, petroleum pipelines, and industrial systems for circulating cool water. PBTCA has the structural features of both phosphoric acid and carboxylic acid groups (Figure 4c), which enable its excellent scale and corrosion inhibition properties [79]. Cao et al. [80] confirmed the usability of HPMA and PBTCA as assistant agents during washing of soil from a waste farmland in the vicinity of a Pb–Zn mine (China) with aqueous plant extracts. Addition of HPMA and PBTCA to plant extracts substantially increased HM removal from soil (Cd by 24%, Pb by 54%, and Zn by 25%) compared to plant extracts without HPMA and PBTCA. Moreover, the addition of HPMA and PBTCA to plant extracts resulted in lower loss of nutrients (NPK) from soil and higher enrichment of soil in organic carbon compared to EDTA.

So far, the usability of the above novel WAs was confirmed at a laboratory scale. Irrespective of the origin and the method of their isolation, WAs from wastes must be analyzed in detail and treated if necessary to be safe for using [65]. It should be mentioned that, despite the high efficiency of soil treatment with these novel WAs, they can be a potential source of soil contamination with pathogenic microorganisms. For example, sewage sludge can contain a wide variety of pathogens including bacteria, viruses, fungi, and eggs of parasites. Thus, sewage sludge free of pathogenic microorganisms should be selected for production of new WAs. To eliminate the potential risk of microbial contamination of soil coming from waste-derived WAs, suitable waste treatment can be used. For example, thermophilic waste composting produces composts free of pathogenic microorganisms. This is because the heat produced by thermophilic bacteria kills pathogenic bacteria. For certainty, prior to selecting the waste from which the WAs are obtained, analyses for pathogenic microorganisms should also be included.

## 4. Treatment of SWSs after Soil Washing/Soil Flushing

Although soil washing/soil flushing ensures high effectiveness of remediation of soils contaminated with HMs, both methods generate secondary fluid waste products (termed SWS) requiring further treatment. Regardless of the type of washing solution used for soil treatment, i.e., weak inorganic acids, weak low-molecular-weight organic acids and/or their salts, chelating agents like EDTA, biosurfactants (e.g., saponin, rhamnolipids), or novel WAs originated from waste materials (DOM, SHS), high HM removal is generally obtained at low pH. This is because the main mechanisms for HM removal from soil under acidic conditions are dissolution and solubilization of specific soil components and HM release. Thus, SWSs are generally of low reactivity, often in the pH range of 2.0–4.0. 

The choice of an appropriate WA is a critical point, not only to guarantee high remediation effectiveness, but also to avoid problems related to SWS final disposal. The management of SWSs, in fact, significantly increases the total cost of soil remediation. Moreover, major environmental concerns can be associated with the use of toxic or non-biodegradable agents, as well as with the use of acidic solutions. Thus, the final stage of soil remediation must include treatment of SWS. However, the treatment method depends on the type of used WA. For example, one of the main problems in EDTA-based soil washing technologies can be separation of EDTA–HM complexes from the SWS, as EDTA and its complexes are toxic and poorly degradable. In the case of more eco-friendly, but more expensive WAs, e.g., saponin biosurfactant, its recovery and reuse should be taken into consideration.

For treatment of SWSs, different physico-chemical methods can be used, including adsorption [81,82,83,84], precipitation [85,86,87,88,89], advanced oxidation processes [90,91,92,93,94,95], and membrane technology [96,97,98]. Although the latest soil remediation tests were focused on the suitability of novel WAs (DOM, HLS, and SHS from different organic wastes), data about their treatment after soil washing/soil flushing are missing. Therefore, in this section, methods of SWS treatment after soil washing with conventional WAs are reviewed. The composition of SWSs is usually complex (e.g., HMs, fine particulates, organic carbon, nutrients), but most studies dealt only with removal of HMs.

### 4.1. Adsorption

Wassay et al. [81] analyzed the efficiency of various adsorbents (granular activated carbon (GAC), granular activated alumina (GAA), and a ferric chloride solution (FCS)) in treating SWS containing HM chelates generated during soil washing with weak organic acids and/or their salts, EDTA, or DTPA. The authors showed that only GAC effectively removed HMs (Cd, Cu, Cr, Hg, Mn, Pb, and Zn) as chelates. In an optimum pH range from 5.4 to 6.9, 97% of Hg was removed, while, in an optimum pH range from 6.9 to 7.7, 78–96% of Cd, Cu, Mn, Pb, and Zn were removed. The efficiency of Cr removal was 77%. Moreover, the brown SWS became colorless after GAC treatment.

Meunier et al. [82] examined the efficiency of natural adsorbents (cocoa shells, cedar bark, pine bark, spruce bark, vermiculite, volcanic rocks) for removing Pb from acidic SWS during soil washing with hydrochloric acid. The SWS contained 45.4 mg Pb/L and had a strong acidic reaction (pH 1.59). Apart from Pb, the SWS also contained Al (39.3 mg/L), Ca (2300 mg/L), Cd (0.061 mg/L), Cu (3.91 mg/L), Fe (11.2 mg/L), Mg (139 mg/L), Ni (0.237 mg/L), and Zn (10.4 mg/L). Cocoa shells were the most efficient sorbent with a maximum sorption capacity of 2.60 mg Pb/g. The removal of Pb from the SWS after 24 h of adsorption with cocoa shells at a dosage of 10 g/L was 47.6%, while it was 30.7% with cedar bark. Kinetic measurements of Pb removal by cocoa shells revealed that sorption equilibrium was obtained after approximately 4 h.

Sullivan et al. [83] found that surfactant-modified zeolite (SMZ) can be a suitable sorbent for As removal from synthetic SWS (pH 12). The maximum sorption of As at 25 °C was 5400 mg/kg, while that at 15 °C decreased to 3150 mg/kg. The As removal by SMZ was attributed to anion exchange with counterions on the surfactant head groups and/or to partitioning of organic carbon–As complexes into the surfactant bilayer. In addition, the SMZ removed up to 97% of dissolved organic carbon from SWS and decolorized it.

Gusiatin [84] demonstrated effective recovery of saponin from SWSs after washing of soils contaminated by As ore processing with clinoptilolite modified by FeCl_3_. Depending on the As content in treated soils (4294–7598 mg/kg), concentration of As removed with saponin varied between 9.5 and 54.6 mg/L. Modified clinoptilolite removed ≥80% of As from saponin effluents at its very high dosage (300 g/L). In addition, recovered and reused saponin, after pH adjustment, removed As from soil with an effectiveness similar to that of the original saponin.

### 4.2. Precipitation

Precipitation is another method proposed for SWS treatment. Hong et al. [85] considered precipitation as a useful way to recover saponin from SWS. At pH 10.7, the precipitation of HMs in SWS was 86% for Cd, 80% for Cu, 90% for Pb, and 91% for Zn. The recovered saponin was able to remove HMs from soil with slightly lower efficiency than the original saponin. This indicates the possibility of subsequent utilization of recovered saponin.

Palma et al. [86] conducted a two-step experiment to recover EDTA from SWS. With initial evaporation and reduction of SWS volume by about 75%, the content of diluted Pb–EDTA was concentrated. This was followed by the acidification of the residual solution and precipitation of EDTA (above 93%). Under alkaline conditions, the HM–EDTA complex can in turn be dissociated, while HMs precipitate as hydroxides.

Lo et al. [87] assessed the feasibility of chemical precipitation for EDTA recovery from SWS after sandy soil washing. In the recovery method, Pb or Zn was firstly dissociated from Pb– or Zn–EDTA complexes through replacement reactions by adding FeCl_3_, and then Pb was removed as phosphate precipitate by adding Na_2_HPO_4_. Finally, Fe (III) was removed as Fe(OH)_3_ precipitates through adding Ca(OH)_2_. As a result, EDTA was recovered as Ca–EDTA. The optimum conditions for EDTA recovery from the Pb–EDTA solution were FeCl_3_:EDTA = 1:1 and Na_2_HPO_4_: EDTA = 2:3, with pH 3.5 after adding Na_2_HPO_4_ and pH 11 after adding Ca(OH)_2_. For Zn–EDTA solution, these conditions were as follows: FeCl_3_:EDTA = 3:2 and Na_2_HPO_4_:EDTA = 4:3, with pH 7.5 after adding Na_2_HPO_4_ and pH 11 after adding Ca(OH)_2_. Under these optimum conditions, 96% of Pb or 83% of Zn was removed from the SWS. Phosphate precipitation and the adsorption of the resulting FePO_4_ or Fe(OH)_3_ precipitates decided the SWS treatment. 

Gao et al. [88] used the precipitation method by adding NaOH to spent saponin solution after washing of industrial sludges to remove HMs. At pH 10.9, the recovery efficiencies of Pb, Ni, and Cr were 89.7%, 91.1%, and 99.1%, respectively. Due to the amphoteric nature of Pb and Cr, their hydroxides were re-dissolved, diminishing the efficiency of HM recovery at high pH (i.e., >11.5). Therefore, the optimum pH for saponin recovery was considered to be about 10.9.

Mukhopadhyay et al. [89] used FeCl_3_ to precipitate As from spent soapnut saponin solution. It was found that saponin could be recovered from the SWS at low dosage of FeCl_3_ (8–10 mg/L) and pH 8. Under these conditions, up to 87% of As was removed through coagulation, flocculation, and precipitation. FeCl_3_ was found to be an effective precipitating agent for saponin recovery from SWS polluted with As.

### 4.3. Advanced Oxidation Processes (AOPs)

In AOPs, ozone, H_2_O_2_, sonification, and ultraviolet (UV) can be used for SWS treatment. Using these methods, recovery of original WA is not possible, because it is degraded to other by-products. Jiraroj et al. [90] studied the degradation of Pb–EDTA, Cd–EDTA, and Zn–EDTA complexes via an H_2_O_2_/UV process in aqueous solution. The authors found that the decomposition of HM–EDTA complexes and HM removal via the H_2_O_2_/UV process depended greatly on the HM nature. Pb–EDTA degradation was accompanied by simultaneous Pb precipitation. Pb–EDTA was decomposed rapidly in acidic solutions, while Pb precipitation was decomposed at pH higher than 6. Cd–EDTA and Zn–EDTA were decomposed rapidly, but the HMs were not precipitated. The major by-products of HM–EDTA degradation were nitrilotriacetic acid (NTA), iminodiacetic acid (IDA), oxalic acid, and nitrate.

Treatment of SWS after soil washing with EDTA was the subject of intensive research conducted by Finžgar and Leštan [91,92,93] and Leštan and Finžgar [94]. Firstly, the authors [91] examined the feasibility of using AOPs (ozone, ozone/sonification, and ozone/UV) to remove HMs and EDTA from SWS. AOP generated –OH for the oxidative decomposition of EDTA–HM complexes. Among tested AOPs, only ozone/UV decomposed EDTA–HM complexes in the SWS. The released Pb and Zn were recovered by their sorption onto commercial Slovakite sorbent (a mixture of natural raw materials: dolomite, diatomite, smectite basaltic tuff, bentonite, alginite, and zeolite) [94]. After such treatment, the concentrations of Pb, Zn, and EDTA in SWS were relatively low: 2.87 ± 1.15 mg/L, 7.58 ± 2.12 mg/L, and 0.012 ± 0.002 mmol/L, respectively. Thus, the use of ozone/UV is a feasible option for SWS treatment from EDTA soil washing. In a further study by Finžgan and Leštan [92], combination of ozone and UV with HM adsorption on Slovakite sorbent allowed obtaining low concentrations of EDTA and Pb in spent EDTA solution. However, the ozone-UV method was efficient only for fairly colorless and non-turbid solutions, which are not typical for soil washing technology. Another practical problem is the high consumption of HM adsorbent and its further management.

In another study, Finžgar and Leštan [93] used an electrochemical advanced oxidation process (EAOP) for treatment of SWS containing HM–EDTA complexes. In EAOP, a boron-doped diamond anode was used for generation of hydroxyl radicals and oxidative decomposition of HM–EDTA complexes at a constant current density (15 mA/cm^2^). The HMs (as insoluble precipitate) were removed from the solution via filtration and electro-deposition on the cathode. Before treatment in the electrolytic cell, the SWS had the following characteristics: pH 8.1, 2057 ± 121 mg EDTA/L, 495 ± 36 mg Pb/L, 119 ± 5 mg Zn/L, 4.3 ± 0.2 mg Cd/L, 303 ± 17 mg Ca/L, and 54 ± 5 mg Fe/L. The EAOP efficiently removed Pb, Zn, Cd, and EDTA from the SWS. After 60 min of contact time with a current density of 25 mA/cm^2^, 99% of Pb and EDTA, 92% of Zn, and 90% of Cd were removed. 

Satyro et al. [95] used TiO_2_ photocatalysis for SWS treatment from EDDS soil washing, containing 7.8 mg Cu/L, 10.5 mg Fe/L, and 5.7 mg Zn/L. The combined photocatalytic process can be used for SWS treatment (Cu < 0.05 mg/L, Fe 0.09 mg/L, and Zn 0.8 mg/L). 

### 4.4. Membrane Technologies

The main objective of membrane technologies is to concentrate the pollutants in SWS as much as possible and to reduce the volume of SWS. An effective membrane technology greatly reduces the volume of SWS and produces pollutant-free SWS that can be reused or discharged. SWS can contain particulates; thus, microfiltration may be used as the first stage of treatment. The soluble pollutants in the filtrate can then be concentrated with another membrane method, e.g., ultrafiltration (UF), nanofiltration (NF), or reserve osmosis (RO).

Volchek et al. [96] employed a combination of microfiltration and nanofiltration for acidic SWS treatment. Microfiltration was used to separate soil particles from the HM-containing SWS, and then the SWS was processed with nanofiltration (to reduce the SWS volume and to recover spent acid). The HMs (Pb, Cu, Zn, Fe, Al, Sr, Na, K, Ba) were subsequently precipitated and removed. The remaining acid can possibly be reused. Under optimum conditions, the rejection of HMs exceeded 95% and the SWS volume could be reduced by 90%. While more than 90% of most HMs were rejected, the acid was not rejected at all, and the permeate had a lower pH than the concentrate. The authors stated that the main challenge associated with the use of nanofiltration is the presence of Fe in SWS at high concentrations. This is because Fe precipitation leads to rapid membrane fouling. To avoid such a problem, the SWS was fortified with fresh acid to maintain a lower pH and to keep Fe in a soluble form. As a result, permeate flux was more stable and the permeate did not require the addition of fresh acid prior to its reuse.

Ortega et al. [97] analyzed the performance of two commercial nanofiltration membranes (Desal5 DK and NF-270) for HM removal from acidic SWS after soil washing with 36 M H_2_SO_4_ (pH 3). Membrane performance was evaluated based on membrane permeability and ionic retention in the tank and permeate. Membranes showed high ion selectivity and good HM rejection (62–100%), higher for divalent than for monovalent HM ions, as well as high TOC removal (70–89%). During the filtration experiments, the dynamic permeability of the Desal5 DK membrane increased, while it decreased for NF-270 membrane. Due to good membrane permeability, ion retention, and acidic resistance, Desal5 DK was considered the most efficient membrane for HM removal. 

Membranes only separate the pollutants from the SWS, without decreasing the hazardous pollutant nature. Thus, other technologies must be used in conjunction with membranes to deal efficiently with pollutants. For HMs, their precipitation followed by landfilling may be an option. Ortega et al. [98] integrated NF and electrochemical treatment as a feasible method for the treatment of acidic SWSs, i.e., HCl (pH 2) and H_2_SO_4_–NaCl (pH 2). NF is able to separate different ions according to their valence, and it has the ability to remove inorganic pollutants through electrostatic interactions between the ions and membranes. According to the authors, high-quality permeate can be obtained with the application of NF. On the other hand, the concentrate can be problematic due to the high pollutant concentration of pollutants. An alternative and effective process to reduce the levels of pollutants in the concentrate is electrochemical treatment. The results showed that the NF membrane (Desal-5) had a high ion-retention rate for both SWSs. Conductivity was reduced by more than 50% (6.1 mS/cm in HCl solution and 144 mS/cm in H_2_SO_4_–NaCl solution). More than 65% of inorganic ions were retained. Higher retention was achieved for SWS containing HCl than H_2_SO_4_–NaCl. In order to maximize the removal of HM ions from the NF concentrate, the solutions containing Cu, Mn, Ni, Pb, and Zn were treated electrochemically using insoluble electrodes (platinum-coated titanium anode and stainless-steel cathode). Greater HM removal was observed in H_2_SO_4_–NaCl solution (approximately 97% for Pb, Mn, and Cu) than in HCl solution (88% Pb, 64% Cu, and 93% Mn).

## 5. Conclusions

Soil pollution with HMs is still an increasing global problem; thus, development of soil remediation methods based on permanent HM removal with soil washing/soil flushing is necessary. Currently, soil washing technology continues to progress toward the use of environmentally friendly, cost-effective, and easily available WAs. Great potential in this area is shown by so-called next-generation WAs isolated from organic waste materials, such as DOM, HLS, or SHS. Currently, the use of these WAs in soil remediation is being intensively investigated, but these investigations mainly focus on the efficiency of HM removal. To create operational and safety guidelines for using novel WAs for soil remediation, future studies should aimed to (1) determine the detailed composition of novel WAs and assess their impact on soil quality in terms of organic matter, carbon sequestration, nutrients content, and microbial contamination, (2) assess their effectiveness in soil treatment under continuous conditions (soil flushing) for potential application in field soils, and (3) perform detailed characterization of SWSs in relation to their further treatment and reuse.

## Figures and Tables

**Table 1:** Ranges of heavy metal (HM) concentrations in soils (in mg/kg) in selected countries.

Source/Location	As	Cd	Cr	Cu	Ni	Pb	Zn	Ref.
Area around Pb and Zn mine (farmland, pasture, mining waste); Spain	–	–	–	–	–	368–18,260	449–9489	[10]
Area around former smelter; Czech Republic	–	5.7	–	21.7	–	1160	233	[11]
Urban area, Pb–Zn–Ag and Cu mines; Australia	–	1.0–9.8	–	34–2730	–	1–2020	49–15,600	[12]
Zinc industrial complex; Iran	–	0.2–18.5	–	–	–	4.1–630	146.6–3066	[13]
Electroplating industry; India	–	–	1172–3240	263–374	234–335		1376–3112	[14]
Metallurgical factory; Zambia	0.04–255	–	–	34–27,410	–	4.0–480	4.0–450	[15]
Industrial and mining complex with battery factory; Kosovo	16–110	0.3–11.8	–	17.8–134	–	53.4–5536	86.1–1553	[16]
Industrial area (e.g., paint, plastic, metal industry); Turkey	1.5–66	0.05–176	10–1161	8–725	–	17–8469	29.5–10,000	[17]
Pb–Zn mining; Zambia	0.04–141	0.01–139	3–110	2–5727	2–74	9–51188	5–91,595	[18]
Smelting industry; Poland	–	–	–	855–13,143	–	585–9181	718–3363	[19]
Metallurgical industry; Mexico	4019	14.4	–	35,582	2063	70	261.4	[20]
Urban soils, Tiexi Industrial District (heavy machinery and manufacturing industry); China	–	0.01–9.6	4.8–207	7.6–430	–	1.9–940	25–1140	[21]

**Table 2:** Summary of soil washing and soil flushing technologies [1,3,22,38]. USD—United States dollars.

Feature	Soil Washing	Soil Flushing
Applicability	Pollutant(s): broad range, e.g., HMs, gasoline, fuel oils, and pesticidesSoils: permeable with low content of clay and organic matter, moderate to high contamination	
	*Site:* ex situ (on site or off site)	*Site*: in situ
	*Scale*: small	*Scale*: wider
	*Status:* regularly practiced	*Status*: limited number of applications
Pre-treatment	Soil excavation is required; reduction of soil volume by physical separation with special equipment (e.g., trommels, attrition or flotation machines, screens, hydrocyclones)	Soil excavation is not required; installation of barriers in ground; injection of washing solution into ground
Soil treatment	Pollutant removal from separated soil fractions by extraction with washing solutions	Pollutant removal by percolation of washing solutions through contaminated zone
Post-treatment	Stabilization and disposal of concentrated soil fraction, treatment of SWSs, pollutant recovery	Treatment of SWSs, groundwater treatment
Advantages	Permanently removes pollutants from soil; a rapid method; effective even for highly polluted soils	Minimal soil disturbance; short to medium treatment time; lower cost
Limitations	Deterioration of soil structure and soil composition (e.g., nutrients removal); a relatively high cost	Effective only for permeable, coarse-textured soils; a potential risk of groundwater pollution
Cost ($USD/ton)	25–300	100–200

SWS: of spent washing solutions.

## Abbreviations

The following abbreviations were used in this manuscript:

AOP	advanced oxidation process
CHA	commercial humic acid
DOM	dissolved organic matter
DTPA	diethylenetriaminepentaacetic acid
EAOP	electrochemical advanced oxidation process
EDDS	ethylenediaminedisuccinic acid
EDTA	ethylenediaminetetraacetic acid
FA	fulvic acid
FCS	ferric chloride solution
GAA	granular activated alumina
GAC	granular activated carbon
GLDA	*N,N*-bis(carboxymethyl)-l-glutamic acid
HA	humic acid
HLS	soluble humic-like substances
HM	heavy metal
HPMA	hydrolytic polymaleic anhydride
HS	humic substances
IDA	iminodiacetic acid
KLS	potassium lignosulfonate
NF	nanofiltration
NOM	natural organic matter
NTA	nitrilotriacetic acid
PBTCA	2-phosphonobutane-1,2,4-tricarboxylic acid
RO	reserve osmosis
SHLA	synthetic humic-like acids
SHS	soluble humic substances
SHS	soluble humic substances
SMZ	surfactant-modified zeolite
SWS	spent washing solution
TOC	total organic carbon
UF	ultrafiltration
WA	washing agent
WPWS	wine-processing waste sludge

## References

[1] Liu L., Li W., Song W., Guo M. (2018). Remediation techniques for heavy metal-contaminated soils: Principles and applicability. Sci. Total Environ..

[2] Kabir E., Ray S., Kim K.-H., Yoon H.-O., Jeon E.-C., Kim Y.S., Cho Y.-S., Yun S.-T., Brown R.J.C. (2012). Current Status of Trace Metal Pollution in Soils Affected by Industrial Activities. Sci. World J..

[3] Gong Y., Zhao D., Wang Q. (2018). An overview of field-scale studies on remediation of soil contaminated with heavy metals and metalloids: Technical progress over the last decade. Water Res..

[4] Shahid M., Khalid S., Abbas G., Shahid N., Nadeem M., Sabir M., Aslam M., Dumat C., Hakeem K.R. (2015). Heavy metal stress and crop productivity. Crop Production and Global Environmental Issues.

[5] Khalid S., Shahid M., Niazi N.K., Murtaza B., Bibi I., Dumat C. (2017). A comparison of technologies for remediation of heavy metal contaminated soils. J. Geochem. Explor..

[6] ATSDR Priority List of Hazardous Substances. https://www.atsdr.cdc.gov/SPL/index.html.

[7] Gorospe J. (2012). Growing Greens and Soiled Soil.

[8] USEPA (2004). Cleaning Up the Nation’s Waste Sites Markets and Technology Trends.

[9] EEA Progress in Management of Contaminated Sites. https://www.eea.europa.eu/data-and-maps/indicators/progress-in-management-of-contaminated-sites-3/assessment.

[10] Rodríguez L., Ruiz E., Alonso-Azcárate J., Rincón J. (2009). Heavy metal distribution and chemical speciation in tailings and soils around a Pb–Zn mine in Spain. J. Environ. Manag..

[11] Janoš P., Vávrová J., Herzogová L., Pilařová V. (2010). Effects of inorganic and organic amendments on the mobility (leachability) of heavy metals in contaminated soil: A sequential extraction study. Geoderma.

[12] Taylor M.P., Mackay A.K., Hudson-Edwards K.A., Holz E. (2010). Soil Cd, Cu, Pb and Zn contaminants around Mount Isa city, Queensland, Australia: Potential sources and risks to human health. Appl. Geochem..

[13] Parizanganeh A., Hajisoltani P., Zamani A. (2010). Assessment of heavy metal pollution in surficial soils surrounding Zinc Industrial Complex in Zanjan-Iran. Procedia Environ. Sci..

[14] Ahlawat Sainger P., Dhankhar R., Sainger M., Kaushik A., Pratap Singh R. (2011). Assessment of heavy metal tolerance in native plant species from soils contaminated with electroplating effluent. Ecotoxicol. Environ. Saf..

[15] Ettler V., Mihaljevič M., Kříbek B., Majer V., Šebek O. (2011). Tracing the spatial distribution and mobility of metal/metalloid contaminants in Oxisols in the vicinity of the Nkana copper smelter, Copperbelt province, Zambia. Geoderma.

[16] Nannoni F., Protano G., Riccobono F. (2011). Fractionation and geochemical mobility of heavy elements in soils of a mining area in northern Kosovo. Geoderma.

[17] Yaylalı-Abanuz G. (2011). Heavy metal contamination of surface soil around Gebze industrial area, Turkey. Microchem. J..

[18] Nakayama S.M.M., Ikenaka Y., Hamada K., Muzandu K., Choongo K., Teraoka H., Mizuno N., Ishizuka M. (2011). Metal and metalloid contamination in roadside soil and wild rats around a Pb–Zn mine in Kabwe, Zambia. Environ. Pollut..

[19] Medyńska-Juraszek A., Kabała C. (2012). Heavy metal pollution of forest soils affected by the copper industry. J. Elem..

[20] Torres L.G., Lopez R.B., Beltran M. (2012). Removal of As, Cd, Cu, Ni, Pb, and Zn from a highly contaminated industrial soil using surfactant enhanced soil washing. Phys. Chem. Earth Parts ABC.

[21] Li X., Liu L., Wang Y., Luo G., Chen X., Yang X., Hall M.H.P., Guo R., Wang H., Cui J. (2013). Heavy metal contamination of urban soil in an old industrial city (Shenyang) in Northeast China. Geoderma.

[22] Dermont G., Bergeron M., Mercier G., Richer-Laflèche M. (2008). Soil washing for metal removal: A review of physical/chemical technologies and field applications. J. Hazard. Mater..

[23] Pearl M. (2007). Understanding Soil Washing. CL AIRE Tech. Bull..

[24] Hubler J., Metz K. Soil Washing. https://www.geoengineer.org/education/web-class-projects/cee-549-geoenvironmental-engineering-winter-2013/assignments/soil-washing.

[25] Anderson W.C. (1993). Soil Washing/Soil Flushing.

[26] Griffiths R.A. (1995). Soil-washing technology and practice. J. Hazar. Mater..

[27] Dekonta Transportable Soil Washing Plant. https://www.dekonta.cz/en/about-us/download.html.

[28] USEPA (1996). A Citizen’s Guide to In Situ Soil Flushing.

[29] Mulligan C., Yong R., Gibbs B. (2001). Surfactant-enhanced remediation of contaminated soil: A review. Eng. Geol..

[30] Khan F.I., Husain T., Hejazi R. (2004). An overview and analysis of site remediation technologies. J. Environ. Manag..

[31] Mao X., Jiang R., Xiao W., Yu J. (2015). Use of surfactants for the remediation of contaminated soils: A review. J. Hazard. Mater..

[32] Boskalis Environmental Enhanced Soil Washing. https://environmental.boskalis.com/activities/enhanced-soil-washing.html.

[33] Water Front Toronto Pilot Soil Recycling Facility Fact Sheet. https://www.waterfrontoronto.ca/nbe/wcm/connect/waterfront/40e110c0-8580-485d-a555-88b10e0c79d0/soil_recycling_pilot_facility_fact_sheet___april_2012___clean_1.pdf?MOD=AJPERES&CACHEID=40e110c0-8580-485d-a555-88b10e0c79d0.

[34] FRTR Remediation Technologies Screening Matrix and Reference Guide, Version 4.0. https://frtr.gov/matrix2/top_page.html.

[35] Ko I., Chang Y.-Y., Lee C.-H., Kim K.-W. (2005). Assessment of pilot-scale acid washing of soil contaminated with As, Zn and Ni using the BCR three-step sequential extraction. J. Hazard. Mater..

[36] Boskalis Environmental. https://environmental.boskalis.com/.

[37] ART Engineering LLC. https://www.art-engineering.com/index.html.

[38] Li C., Zhou K., Qin W., Tian C., Qi M., Yan X., Han W. (2019). A Review on Heavy Metals Contamination in Soil: Effects, Sources, and Remediation Techniques. Soil Sediment Contam..

[39] Liu Q., Deng Y., Tang J., Chen D., Li X., Lin Q., Yin G., Zhang M., Hu H. (2019). Potassium lignosulfonate as a washing agent for remediating lead and copper co-contaminated soils. Sci. Total Environ..

[40] Ali M., Mindari W. (2016). Effect of Humic Acid on Soil Chemical and Physical Characteristics of Embankment. MATEC Web Conf..

[41] Damian G.E., Micle V., Sur I.M. (2019). Mobilization of Cu and Pb from multi-metal contaminated soils by dissolved humic substances extracted from leonardite and factors affecting the process. J. Soils Sediment.

[42] Tsang D.C.W., Olds W.E., Weber P. (2013). Residual leachability of CCA-contaminated soil after treatment with biodegradable chelating agents and lignite-derived humic substances. J. Soils Sediment.

[43] Meng F., Yuan G., Wei J., Bi D., Ok Y.S., Wang H. (2017). Humic substances as a washing agent for Cd-contaminated soils. Chemosphere.

[44] Conte P., Agretto A., Spaccini R., Piccolo A. (2005). Soil remediation: Humic acids as natural surfactants in the washings of highly contaminated soils. Environ. Pollut..

[45] Gusiatin Z.M. (2018). Novel and Eco-Friendly Washing Agents to Remove Heavy Metals from Soil by Soil Washing. Environ. Anal. Ecol. Stud..

[46] Kulikowska D., Klik B.K., Gusiatin Z.M., Hajdukiewicz K. (2019). Characteristic of humic substances from municipal sewage sludge: A case study. Desalin. Water Treat..

[47] Li H., Li Y., Zou S., Li C. (2014). Extracting humic acids from digested sludge by alkaline treatment and ultrafiltration. J. Mater. Cycles Waste Manag..

[48] Klik B., Kulikowska D., Gusiatin Z.M., Pasieczna-Patkowska S. (2020). Washing agents from sewage sludge: Efficiency of Cd removal from highly contaminated soils and effect on soil organic balance. J. Soils Sediments.

[49] Feng C., Zhang S., Li L., Wang G., Xu X., Li T., Zhong Q. (2018). Feasibility of four wastes to remove heavy metals from contaminated soils. J. Environ. Manag..

[50] Borggaard O.K., Hansen H.C.B., Holm P.E., Jensen J.K., Rasmussen S.B., Sabiene N., Steponkaite L., Strobel B.W. (2009). Experimental Assessment of Using Soluble Humic Substances for Remediation of Heavy Metal Polluted Soils. Soil Sediment Contam..

[51] Lin K.-Y., Chen Y.-M., Chen L.-F., Wang M.-K., Liu C.-C. (2017). Remediation of Arsenic-Contaminated Soil Using Alkaline Extractable Organic Carbon Solution Prepared from Wine-Processing Waste Sludge. Soil Sediment Contam..

[52] Liu C.C., Wang M.K., Chiou C.S., Li Y.S., Yang C.Y., Lin Y.A. (2009). Biosorption of chromium, copper and zinc by wine-processing waste sludge: Single and multi-component system study. J. Hazard. Mater..

[53] Liu C.C., Lin Y.C. (2013). Reclamation of copper-contaminated soil using EDTA or citric acid coupled with dissolved organic matter solution extracted from distillery sludge. Environ. Pollut..

[54] Liu C.-C., Chen G.-B. (2013). Reclamation of cadmium-contaminated soil using dissolved organic matter solution originating from wine-processing waste sludge. J. Hazard. Mater..

[55] Chen Y.-M., Lin W.-H., Lin Y.-A., Liu C.-C., Wang M.-K. (2014). Remediation of lead-contaminated soil using dissolved organic carbon solutions prepared by wine-processing waste sludge. Geoderma.

[56] Wu J., Zhao Y., Zhao W., Yang T., Zhang X., Xie X., Cui H., Wei Z. (2017). Effect of precursors combined with bacteria communities on the formation of humic substances during different materials composting. Bioresour. Tech..

[57] Guo X., Liu H., Wu S. (2019). Humic substances developed during organic waste composting: Formation mechanisms, structural properties, and agronomic functions. Sci. Total Environ..

[58] Gusiatin Z.M., Kulikowska D. (2015). Influence of compost maturation time on Cu and Zn mobility (MF) and redistribution (IR) in highly contaminated soil. Environ. Earth Sci..

[59] Pérez-Esteban J., Escolástico C., Masaguer A., Moliner A. (2012). Effects of sheep and horse manure and pine bark amendments on metal distribution and chemical properties of contaminated mine soils. Eur. J. Soil Sci..

[60] Piccolo A., Spaccini R., De Martino A., Scognamiglio F., di Meo V. (2019). Soil washing with solutions of humic substances from manure compost removes heavy metal contaminants as a function of humic molecular composition. Chemosphere.

[61] Chiang P.-N., Tong O.-Y., Chiou C.-S., Lin Y.-A., Wang M.-K., Liu C.-C. (2016). Reclamation of zinc-contaminated soil using a dissolved organic carbon solution prepared using liquid fertilizer from food-waste composting. J. Hazard. Mater..

[62] Li H., Li Y., Li C. (2017). Evolution of humic substances during anaerobic sludge digestion. Environ. Eng. Manag. J..

[63] Jansen B., Nierop K.G.J., Verstraten J.M. (2003). Mobility of Fe(II), Fe(III) and Al in acidic forest soils mediated by dissolved organic matter: Influence of solution pH and metal/organic carbon ratios. Geoderma.

[64] Fan C., Zhang Y. (2018). Environmentally friendly remediation of lead/cadmium co-contaminated loess soil in northwestern China using a humificated straw solution. Environ. Sci. Pollut. Res..

[65] Borggaard O.K., Holm P.E., Strobel B.W. (2019). Potential of dissolved organic matter (DOM) to extract As, Cd, Co, Cr, Cu, Ni, Pb and Zn from polluted soils: A review. Geoderma.

[66] Strobel B.W. (2001). Influence of vegetation on low-molecular-weight carboxylic acids in soil solution—A review. Geoderma.

[67] Antoniadis V., Levizou E., Shaheen S.M., Ok Y.S., Sebastian A., Baum C., Prasad M.N.V., Wenzel W.W., Rinklebe J. (2017). Trace elements in the soil-plant interface: Phytoavailability, translocation, and phytoremediation–A review. Earth Sci. Rev..

[68] Gusiatin Z.M. (2014). Tannic acid and saponin for removing arsenic from brownfield soils: Mobilization, distribution and speciation. J. Environ. Sci..

[69] Liu Z., Li Z., Zhong H., Zeng G., Liang Y., Chen M., Wu Z., Zhou Y., Yu M., Shao B. (2017). Recent advances in the environmental applications of biosurfactant saponins: A review. J. Environ. Chem. Eng..

[70] Fukuchi S., Fukushima M., Nishimoto R., Qi G., Sato T. (2012). Fe-loaded zeolites as catalysts in the formation of humic substance-like darkcoloured polymers in polycondensation reactions of humic precursors. Clay Miner..

[71] Yang T., Hodson M.E. (2019). Investigating the use of synthetic humic-like acid as a soil washing treatment for metal contaminated soil. Sci. Total Environ..

[72] Polymer Properties Database, Lignin and Its Derivatives. http://polymerdatabase.com/polymer%20classes/Lignin%20type.html.

[73] Gusiatin Z.M., Kulikowska D., Klik B. (2017). Suitability of humic substances recovered from sewage sludge to remedy soils from a former as mining area—A novel approach. J. Hazard. Mater..

[74] Kulikowska D., Gusiatin Z.M., Bułkowska K., Klik B. (2015). Feasibility of using humic substances from compost to remove heavy metals (Cd, Cu, Ni, Pb, Zn) from contaminated soil aged for different periods of time. J. Hazard. Mater..

[75] Kulikowska D., Gusiatin Z.M., Bułkowska K., Kierklo K. (2015). Humic substances from sewage sludge compost as washing agent effectively remove Cu and Cd from soil. Chemosphere.

[76] Rasmussen S.B., Jensen J.K., Borggaard O.K. (2015). A laboratory test of NOM-assisted remediation of arsenic and copper contaminated soils. J. Environ. Chem. Eng..

[77] Akzo Nobel (2010). Dissolvine^®^ GL Technical brochure.

[78] Wang G., Zhang S., Xu X., Zhong Q., Zhang C., Jia Y., Li T., Deng O., Li Y. (2016). Heavy metal removal by GLDA washing: Optimization, redistribution, recycling, and changes in soil fertility. Sci. Total Environ..

[79] Shandong Taihe Water Treatment Technologies, Co. 2-Phosphonobutane-1,2,4-Tricarboxylic Acid (PBTCA). http://thwater.net/01-PBTCA.htm.

[80] Cao Y., Zhang S., Wang G., Li T., Xu X., Deng O., Zhang Y., Pu Y. (2017). Enhancing the soil heavy metals removal efficiency by adding HPMA and PBTCA along with plant washing agents. J. Hazard. Mater..

[81] Wasay S.A., Barrington S., Tokunaga S. (1999). Efficiency of GAC for Treatment of Leachate from Soil Washing Process. Water Air Soil Pollut..

[82] Meunier N., Blais J.-F., Tyagi R.D. (2002). Selection of a natural sorbent to remove toxic metals from acidic leachate produced during soil decontamination. Hydrometallurgy.

[83] Sullivan E.J., Bowman R.S., Legiec I.A. (2003). Sorption of arsenic from soil-washing leachate by surfactant-modified zeolite. J. Environ. Qual..

[84] Gusiatin Z.M. (2015). Fe-modified Clinoptilolite is Effective to Recover Plant Biosurfactants Used for Removing Arsenic from Soil. Clean Soil Air Water.

[85] Hong K.-J., Tokunaga S., Kajiuchi T. (2002). Evaluation of remediation process with plant-derived biosurfactant for recovery of heavy metals from contaminated soils. Chemosphere.

[86] Di Palma L., Ferrantelli P., Merli C., Biancifiori F. (2003). Recovery of EDTA and metal precipitation from soil flushing solutions. J. Hazard. Mater..

[87] Lo I.M., Zhang W. (2005). Study on Optimal Conditions for Recovery of EDTA from Soil Washing Effluents. J. Environ. Eng..

[88] Gao L., Kano N., Sato Y., Li C., Zhang S., Imaizumi H. (2012). Behavior and Distribution of Heavy Metals Including Rare Earth Elements, Thorium, and Uranium in Sludge from Industry Water Treatment Plant and Recovery Method of Metals by Biosurfactants Application. Bioinorg. Chem. Appl..

[89] Mukhopadhyay S., Mukherjee S., Hashim M.A., Sen Gupta B. (2015). Application of colloidal gas aphron suspensions produced from Sapindus mukorossi for arsenic removal from contaminated soil. Chemosphere.

[90] Jiraroj D., Unob F., Hagège A. (2006). Degradation of Pb–EDTA complex by a H2O2/UV process. Water Res..

[91] Finžgar N., Leštan D. (2006). Advanced Oxidation for Treatment of Aqueous Extracts from EDTA Extraction of Pb and Zn Contaminated Soil. J. Environ. Eng..

[92] Finžgar N., Leštan D. (2006). Heap leaching of Pb and Zn contaminated soil using ozone/UV treatment of EDTA extractants. Chemosphere.

[93] Finzgar N., Lestan D. (2008). The two-phase leaching of Pb, Zn and Cd contaminated soil using EDTA and electrochemical treatment of the washing solution. Chemosphere.

[94] Lestan D., Finzgar N. (2007). Leaching of Pb Contaminated Soil using Ozone/UV Treatment of EDTA Extractants. Sep. Sci. Technol..

[95] Satyro S., Race M., Marotta R., Dezotti M., Guida M., Clarizia L. (2017). Photocatalytic processes assisted by artificial solar light for soil washing effluent treatment. Environ. Sci. Pollut. Res..

[96] Volchek K., Velicogna D., Obenauf A., Somers A., Wong B., Tremblay A.Y. (2002). Novel applications of membrane processes in soil cleanup operations. Desalination.

[97] Ortega L.M., Lebrun R., Blais J.-F., Hausler R. (2008). Removal of metal ions from an acidic leachate solution by nanofiltration membranes. Desalination.

[98] Ortega L.M., Lebrun R., Blais J.-F., Hausler R., Drogui P. (2008). Effectiveness of soil washing, nanofiltration and electrochemical treatment for the recovery of metal ions coming from a contaminated soil. Water Res..

